# Electrophysiological Responses to Alcohol Cues Are Not Associated with Pavlovian-to-Instrumental Transfer in Social Drinkers

**DOI:** 10.1371/journal.pone.0094605

**Published:** 2014-04-14

**Authors:** Jasna Martinovic, Andrew Jones, Paul Christiansen, Abigail K. Rose, Lee Hogarth, Matt Field

**Affiliations:** 1 School of Psychology, University of Aberdeen, Aberdeen, United Kingdom; 2 Department of Psychological Sciences, University of Liverpool, Liverpool, United Kingdom; 3 UK Centre for Tobacco and Alcohol Studies, Liverpool, United Kingdom; 4 School of Psychology, University of Exeter, Exeter, United Kingdom; Erasmus University Rotterdam, Netherlands

## Abstract

Pavlovian to Instrumental Transfer (PIT) refers to the behavioral phenomenon of increased instrumental responding for a reinforcer when in the presence of Pavlovian conditioned stimuli that were separately paired with that reinforcer. PIT effects may play an important role in substance use disorders, but little is known about the brain mechanisms that underlie these effects in alcohol consumers. We report behavioral and electroencephalographic (EEG) data from a group of social drinkers (n = 31) who performed a PIT task in which they chose between two instrumental responses in pursuit of beer and chocolate reinforcers while their EEG reactivity to beer, chocolate and neutral pictorial cues was recorded. We examined two markers of the motivational salience of the pictures: the P300 and slow wave event-related potentials (ERPs). Results demonstrated a behavioral PIT effect: responding for beer was increased when a beer picture was presented. Analyses of ERP amplitudes demonstrated significantly larger slow potentials evoked by beer cues at various electrode clusters. Contrary to hypotheses, there were no significant correlations between behavioral PIT effects, electrophysiological reactivity to the cues, and individual differences in drinking behaviour. Our findings are the first to demonstrate a PIT effect for beer, accompanied by increased slow potentials in response to beer cues, in social drinkers. The lack of relationship between behavioral and EEG measures, and between these measures and individual differences in drinking behaviour may be attributed to methodological features of the PIT task and to characteristics of our sample.

## Introduction

Instrumental and Pavlovian conditioning processes contribute to drug self-administration and ultimately the development of substance use disorders. Drug-seeking behavior is reinforced by the pharmacological actions of drugs of abuse, either because those drugs produce pleasurable consequences [1], or because they alleviate negative states such as those that occur during drug withdrawal [2]. This instrumental conditioning process develops synchronously with a Pavlovian conditioning process, in which repeated experience of the rewarding effects of drugs of abuse is consistently paired with environmental drug-related cues, such as the sight and smell of beer. After multiple pairings those cues are able to evoke conditioned responses such as subjective craving, drug anticipation, physiological arousal, and behavioral approach [3], [4] Although Pavlovian and instrumental responses develop independently, their interaction is known as Pavlovian to instrumental transfer (PIT). This refers to the behavioral phenomenon of increased instrumental responding for a reinforcer when in the presence of conditioned stimuli (CS) that were previously paired with that reinforcer.

On the basis of research with laboratory animals, the most well-supported explanation for PIT effects is that a Pavlovian cue (that is predictive of a particular outcome) retrieves a belief that a particular response-outcome association has a stronger contingency, and that the instrumental response is more likely to be reinforced [5]. In the context of addiction, this means that a drug-related cue evokes an expectation of the drug outcome, which in turn activates instrumental responses that have previously led to that outcome in that context [6]. In a series of studies, Hogarth and colleagues demonstrated PIT effects in cigarette smokers, who increased their instrumental responding for cigarettes compared to chocolate in the presence of a cigarette cue, but they increased their responding for chocolate at the expense of cigarettes when exposed to a chocolate cue [7]–[9]. Interestingly, these PIT effects were maintained even when the reinforcer had been devalued or instrumental responses extinguished. For example, in one study [7] the authors were able to train and then extinguish an instrumental response for tobacco, but subsequent presentation of tobacco cues led to reinstatement of the instrumental response for tobacco. Another study [9] demonstrated that devaluation of rewards (cigarettes and chocolate) through satiety led to suppression of instrumental responding for those reinforcers, but it did not attenuate PIT effects. These findings suggest that although the PIT effect involves retrieval of a representation of the expected reinforcer, this representation does not encode the current incentive value of the reinforcer [10]. Therefore, Pavlovian cues appear to evoke instrumental transfer effects automatically, irrespective of the strength of motivation to consume the drug at the time and also regardless of the drug user' severity of dependence [7], [9]. The implication is that PIT may play an important role in drug-seeking behaviour and in relapse to drug-taking after a period of abstinence [11], which is often triggered in response to drug cues [4] long after the (former) drug user would be considered as dependent and when they are no longer motivated to consume the drug (see [12]).

Previous animal and human studies have characterised the patterns of brain activation that underlie PIT effects produced by natural rewards, and have consistently revealed that the striatum, amygdala, orbitofrontal cortex and mediodorsal thalamus are involved [13]. Other studies have used electroencephalography (EEG) to examine electrophysiological indices of drug cue reactivity and these studies suggest that event-related potentials (ERPs) can provide a sensitive measure of brain activity when registering the motivational salience of drug-related pictorial cues [14]. In a recent meta-analysis, Littel and colleagues [15] identified the P300 and subsequent slow wave potential (SP; also known as the sustained Late Positive Potential or LPP) as ERP components which are reliably enhanced in substance users when they view substance-related cues. The P300 is a transient positive deflection maximal at medial central and parietal sites that generally occurs between 300–800 ms after stimulus presentation, whereas the SP is a sustained continuation of the P300 that lasts for several seconds. The amplitudes of both components are modulated by the evaluation and attentional capture of task-relevant and motivationally-relevant stimuli [16]. Littel and colleagues [15] demonstrated a robust medium effect size for an elevated P300 and SP in participants with substance use disorders that was elicited by drug-related cues compared to neutral cues. As well as participants with substance use disorders, a similar pattern of results is seen in non-dependent substance users, such as social drinkers [17], [18]. Therefore, given that P300 and SP are indices of the motivational and attentional salience of drug-related cues, we predicted that the amplitude of these ERP components in response to alcohol-related cues would be related to the degree of enhancement of instrumental response for alcohol when such cues are presented, i.e. behavioral PIT effects.

In our study, participants first learned to make one instrumental response to attempt to win beer, and a different instrumental response to attempt to win chocolate. Participants then completed a PIT task in which they again selected an instrumental response but this time a picture of beer, chocolate, or a neutral stimulus was displayed before participants selected their response. Electrophysiological activity was analysed during this pre-response period. Our hypotheses were as follows. Firstly, behavioral data would reveal a PIT effect, in that participants would make the instrumental response for beer more frequently when a beer picture was presented, compared to when the neutral stimulus was presented. Secondly, amplitudes of P300 and SP would be significantly greater in response to both beer and chocolate pictures (relative to a neutral stimulus), reflecting the increased motivational salience of beer and chocolate pictures in our sample of participants (who drank beer and ate chocolate regularly). Our third and most important hypothesis related to the inter-relationships between behavioral PIT effects and the amplitudes of these ERP components. We predicted that amplitudes of P300 and SP in response to beer cues (relative to their amplitudes in response to neutral cues) would be positively correlated with the effect of those beer cues on instrumental responding for beer. Finally, we predicted that individual differences in typical alcohol consumption, hazardous drinking and alcohol craving would be significantly positively correlated with the magnitude of event-related potentials that were evoked by alcohol-related cues.

## Methods

### Ethics statement

Ethical approval was granted by the University of Liverpool Research Ethics Committee, and all participants provided informed written consent.

### Participants

Forty participants (20 male) with a mean age of 22.68 years (±3.81) were recruited for the study. Inclusion criteria were: consumption of alcohol (more than one unit; one unit  = 8 g alcohol) and chocolate (more than one standard bar) at least once per week; no history of any neurological/neuropsychiatric disorders; aged 18–30; and right-handedness. Participants were asked to refrain from consuming caffeine for two hours and to abstain from alcohol for the entire day, before attending the laboratory. Thirty-one participants (15 male, mean age 22.57±3.84) remained in the final sample, after the removal of participants with excessive EEG artifacts and participants whose data was not properly recorded (see EEG analysis section below).

### Materials

The concurrent instrumental training and PIT behavioral tasks are based on those described elsewhere [9].

### Concurrent instrumental training

The purpose of the concurrent instrumental training task was to establish two instrumental responses, one for each reinforcer. Each trial began with a white fixation cross that was presented for 1000 ms in the centre of the computer screen. After offset, participants were prompted to ‘press a key’ to win. They were instructed to press the ‘m’ key to attempt to win beer, or the ‘b’ key to attempt to win chocolate (keys were re-labeled to indicate which reward they signified). On each trial, one of the responses (beer or chocolate) was randomly selected to be reinforced: if participants pressed the appropriate key on that trial, they received feedback (‘you win a beer point’ or ‘you win a chocolate point’). If they did not press the correct key, the feedback stated ‘you win nothing’. Over the course of this training block, each response was selected for reinforcement on 50% of trials. There were three sub-blocks each containing 8 trials (4 in which the beer response was reinforced, 4 in which the chocolate response was reinforced). At the end of each sub-block participants were given feedback on how many beer and chocolate points they had won. All responses were made using the right hand.

There was an inter-trial interval (ITI) of 1500 ms. During the ITI a small blue cross was presented in the centre of the screen. Participants were asked to blink only during this cross. Although no EEG was recorded during concurrent training, this served to familiarize participants with blinking during this specific period in the stimulus presentation sequence, in order to reduce the number of blinking artifacts during the subsequent PIT phase.

### Pavlovian to instrumental transfer (PIT)

For the PIT task, a white fixation cross was presented in the centre of the screen for a variable period of 700, 800, 900 or 1000 ms. This was then replaced by a picture (all 75 mm×75 mm) of Becks beer, Diary Milk chocolate or a grey square (which served as a neutral stimulus) for 1500 ms. Immediately afterwards, participants were prompted to ‘press a key’ which meant that they should make one of the instrumental responses that they had learned during the concurrent instrumental training phase. If participants responded too quickly (i.e. before they were prompted to press a key) they were informed they had responded too fast and had won nothing. As with the concurrent training, each key had a 50% chance of yielding its respective reinforcer. During the 1500 ms inter-trial interval, the blue cross was presented again and participants were instructed that they could blink only during this period, in order to reduce artifacts. Unlike in the concurrent training phase participants were not informed if they had won beer or chocolate points, or if they had won nothing, at the end of each trial. This was to prevent participants from forming new stimulus-response associations during the PIT phase, which would have contaminated PIT effects.

Instructions given to participants before the PIT task were deliberately vague. Participants were informed that pictures would be presented before the prompt to ‘press a key’ but they were not told what the pictures would depict (i.e. pictures of beer and chocolate). It was never implied that pictures were informative as to which response would be reinforced on that trial, but the reality (that there was no contingency between the type of picture that was presented and the response that would be reinforced on that trial) was not made explicit either.

During an initial practice block of 12 trials, four trials of each picture type (beer, chocolate or neutral) were presented. The main blocks of the tasks comprised five blocks of 60 trials each, with 20 of each picture type, during which EEG was recorded continuously. Participants received feedback on the number of beer and chocolate points won at the end of each block of 60 trials.

In both tasks, beer and chocolate ‘points’ were awarded, rather than giving actual beer or chocolate for consumption at the end of each trial. This is because repeated ingestion of either substance during the task may have led to satiety and resulted in devaluation of that reward. Furthermore, previous studies suggest that providing ‘points’ for specific reinforcers is sufficient to evoke a specific expectancy for that reinforcer on a trial-by-trial basis [8], [19], [20]. The tasks were programmed using Inquisit 3.0 (Millisecond Software, 2011).

### Procedure

Experimental sessions took place in EEG laboratories in the Department of Psychological Sciences, University of Liverpool, between the hours of 12 and 6pm. After providing informed consent, participants completed a two-week timeline follow back alcohol consumption diary [21], the Alcohol Use Disorders Identification Test [22] and the short version of the Desires for Alcohol Questionnaire [23]. Following completion of these questionnaires participants were informed they would be playing a game in which they could win beer and chocolate to take home with them at the end of the study, by accumulating points during computer tasks. In order to reinforce participants' beliefs that they would actually win these rewards, bottles of Becks and bars of Dairy Milk were placed around the lab (but not in the EEG chamber itself) and were explicitly pointed out by the experimenters, a procedure that we have adopted in previous studies [[24] [19]].

Participants were then fitted with the electrode cap and seated in a sound attenuated chamber approximately 150 cm from the computer screen. They were instructed to use their right hand only for responses and told to blink only when the blue fixation cross was presented. They then completed the concurrent training task, followed by the PIT task. They could rest for short periods in between blocks of the PIT task, during presentation of feedback about the number of beer and chocolate points that they had won. The entire experimental session lasted approximately 90 minutes. Following this, participants were fully debriefed and told they would not receive beer or chocolate. Instead, they received music vouchers or course credit.

### Data Acquisition, Reduction and Analysis

In accordance with previous studies, behavioral PIT effects were calculated by contrasting the number of instrumental responses for beer on trials when pictures of beer were shown, in comparison to trials in which pictures of chocolate, or the neutral stimulus, were shown.

EEG data was continuously recorded at a sampling rate of 512 Hz from 64 locations from the international 10/20 system [25] using active Ag-AgCl electrodes (Biosemi ActiveTwo amplifier system, Biosemi, Amsterdam, Netherlands). The Biosemi system replaces traditional ‘ground’ electrodes with two active electrodes, the Common Mode Sense (CMS) and Driven Right Leg (DRL). CMS acts as a recording reference and DRL serves as ground [26], [27]. EEG data processing was performed using the EEGlab toolbox [28] for Matlab (The Mathworks, Inc, Natick, Massachusetts). Continuous EEG was reduced to epochs of 2000 ms, comprising the period between 500 ms before and 1500 ms during picture presentation. Data was low-pass filtered at 40 Hz using EEGlab's Butterworth filter. Vertical EOG responses were recorded in order to exclude trials with blinks. FASTER (Fully Automated Statistical Thresholding for EEG artifact Rejection) plug-in for EEGlab [29] was initially used to remove trials with gross artifacts. Data was referenced to Fz before FASTER was used, as suggested [29], while all other steps were completed using the average reference. Correction of artifacts was continued with the independent component analysis (ICA) incorporated in the ADJUST plug-in for EEGlab [30]. ADJUST identifies artifactual ICA components through statistical properties characteristic of vertical and horizontal eye movements, blinks or noisy electrodes. Following this step FASTER was again used to detect and interpolate contaminated channels. Finally, the efficiency of these two automated artifact rejection and correction methods was verified by visual inspection. Nine participants were removed from further analyses, two due to technical problems during the recording and seven due to excessive artifacts in the data (for these participants, more than 33% of their trials were rejected). As described in the Participants section, the final sample comprised 31 participants. The average trial rejection rate for these participants was 9.77%.

To constrain our ERP analysis, we used an approach that is somewhat novel for the field of substance use disorders, but has been widely used in psychophysiology: the examination of topographic changes in EEG activity (for overviews, see [31],[32]; for examples of studies that use it see [33]–[34]). This approach considers whole-scalp EEG activity elicited by a stimulus as a finite set of alternating spatially stable activation patterns, which reflect a succession of information processing stages. The evolution of whole-scalp activity can be assessed over time in order to see how it differs between experimental conditions that impose different information processing demands. Differences in topographic patterns of activity between conditions are assessed using Cartool software (http://sites.google.com/site/cartoolcommunity/). There are two main reasons why this approach is more objective than the more traditional assessment of amplitudes and/or latencies of a set of pre-defined ERP components. First, it takes into consideration the entire time course of activity and the entire pattern of activation across the scalp, by testing the global field power from all electrodes (for more detail on global field power as a measure of whole-scalp activity at each time-point, see [35]). Second, this approach is able to detect not only differences in amplitude, but also differences in underlying sources of activity, because maps that are confirmed to be both spatially and temporally different must necessarily be the product of a different set of generators. However, we emphasize that the analysis of topography changes is not incompatible with the analysis of traditional ERPs. On the contrary, the time-windows of significant topographical differences can be used to define windows for testing of amplitudes of pre-selected ERP components [35], which removes biases inherent in traditional ERP amplitude analyses [36] but still allows for comparisons with previous ERP literature. This is the approach we have taken here, using topographical analysis to make our analysis more objective by focusing on the time windows in which significant differences between stimulus-elicited activities are found during the P300 and the SP periods.

As recommended [31], topographical differences were tested through a non-parametric randomization test known as TANOVA (Topographic ANOVA). TANOVA tests for differences in global dissimilarity of EEG activity between two conditions by assessing if the topographies are significantly different from each other on a timepoint-by-timepoint basis. TANOVAs were conducted to assess differences in activation patterns during presentation of beer vs. chocolate, beer vs. neutral and chocolate vs. neutral images during the PIT phase. TANOVA is sufficient for indicating the time-windows of interest for ERP analyses. However, further assessment of topographical differences also indicates if the observed effects stem from the same sources or from different sources. For the sake of brevity, and as our hypotheses mainly concern ERP effects, we present further methodological details and results of these topographical analyses in Material S1.

**Figure 1:**
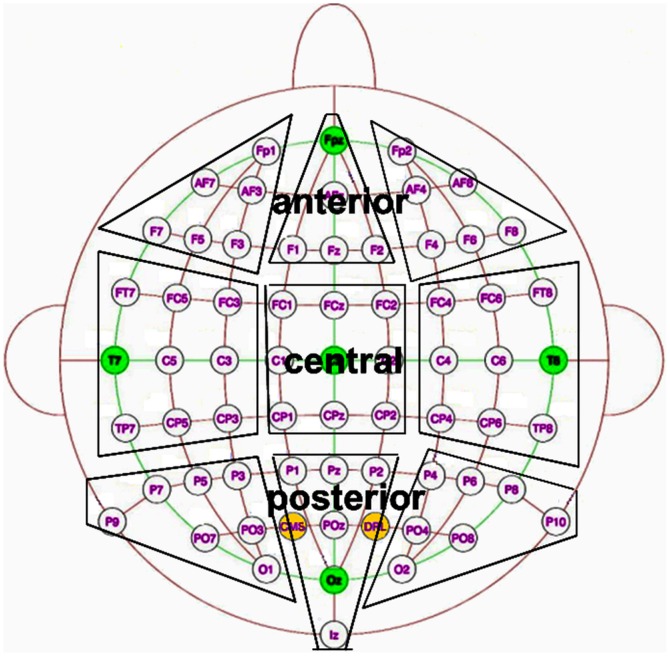
Electrode sites of interest. Electrode sites of interest: anterior, central and posterior on the left, midline and the right side of the head. The two active electrodes that are shown are CMS (Common Mode Sense) which acts as a recording reference and DRL (Driven Right Leg) which serves as a ground in the Biosemi ActiveTwo system.

Event-related potential (ERP) amplitudes were calculated from nine sets of electrode sites (anterior, central and posterior; left, midline and right; see Figure 1). Differences in amplitudes were contrasted during the time periods in the P300 and/or SP windows indicated by the TANOVA to be significantly different, using a 200 ms pre-stimulus period as baseline. A 3 (picture type: beer, chocolate, neutral)×3 (electrode laterality: left, midline, right)×3 (electrode position: anterior, central, posterior) repeated measures ANOVA was performed on mean amplitudes in the chosen windows. Main effects or interactions involving the picture type variable were of particular interest. In cases where such effects were found, Bonferroni-corrected, Greenhouse-Geiser corrected post-hoc ANOVAs, followed up by post-hoc Bonferroni corrected t-tests, were used to assess if the difference was driven by a beer/neutral or chocolate/neutral contrast or if they were driven by the difference between the two rewarding stimuli themselves.

In order to investigate whether individual differences in drinking behavior would moderate the behavioral PIT effect or its presumed electrophysiological correlates, we divided our sample into relatively high vs. relatively low hazardous drinking groups based on a median split of AUDIT scores. We then re-ran our ANOVAs with the addition of this as a further between-subjects variable.

Finally, in order to relate the patterns of brain activity to behavioral PIT effects and individual differences in drinking behavior, subtracted scores were computed between ERP amplitudes that were found to discriminate between beer vs. chocolate and beer vs. the neutral stimulus. These were then correlated with beer-related behavioral PIT effects, and self-reported alcohol consumption, hazardous drinking, and craving (units of alcohol consumed per week, AUDIT and DAQ scores, respectively).

## Results

### Participant characteristics

Participants consumed an average of 47.48 units of alcohol (SD±25.75) over the 14 days prior to taking part in the study. The mean total score on the DAQ was 2.69 (±0.96), and the mean score on the AUDIT was 13.16 (±5.40). Eighty one percent of participants had a score of 8 or higher on the AUDIT, the cutoff score indicative of hazardous drinking [22]. Participants were split into groups based on a median split of AUDIT scores (median  = 13), which resulted in 16 participants in the low AUDIT group and 15 participants in the high AUDIT group.

### Behavioral Pavlovian to instrumental transfer effects

**Figure 2:**
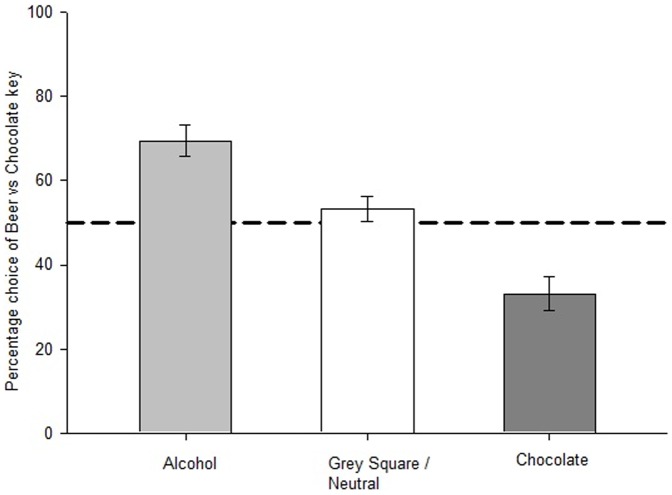
Mean percentage instrumental responses on the beer key following each cue type. Mean percentage instrumental responses on the beer key (±standard error of the mean) following beer, chocolate and neutral grey square during the PIT task.

We performed a repeated-measures ANOVA to investigate the percentage of responses on the beer key during presentation of the different types of picture (beer, chocolate, or neutral grey square). This revealed a significant main effect of picture type (F(2, 60) = 30.35, p<.01) illustrated in Figure 2. Planned comparisons revealed that participants responded for beer more frequently when beer pictures were presented compared to when chocolate (t(30) = 5.80, p<.01, *d* = 1.66) and neutral pictures (t(30) = 4.98, p<.01, *d* = 0.87) were presented, i.e. a ‘beer PIT’ effect. Furthermore, responses on the beer key were significantly lower during presentation of the chocolate picture compared to the grey square picture (t(30) = −5.08, p<.01, *d* = 1.01). The latter is a ‘chocolate PIT’ effect: presentation of the chocolate picture leads to more responses on the chocolate key rather than the beer key.

To investigate whether participants' drinker status (above or below the median on the AUDIT) moderated the behavioral PIT effect, we repeated this analysis with the addition of group as a between-subjects variable. The main effect of picture type remained significant (F(2, 58) = 31.60, p<.01). However, the main effect of drinker status (F(1, 29) = .28, p = .60), and the drinker status x picture type interaction (F(2, 58) = 1.21, p = .31) were not statistically significant. Therefore, more hazardous drinkers (those who scored above the sample median on the AUDIT) did not make more responses on the beer key overall, and the magnitude of the PIT effect did not differ between the two groups.

### EEG

#### TANOVA

**Figure 3:**
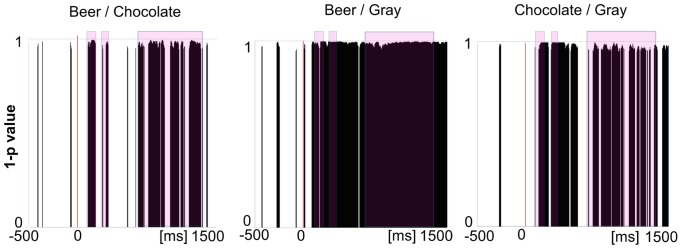
Time points of significant differences in EEG activity for the three main contrasts. Time points of significant differences in EEG activity for the three main contrasts (beer vs. chocolate, beer vs. grey and chocolate vs. grey) as indicated by the TANOVA analysis, depicting 1 minus p-value across time. Significant p values are plotted. The colored squares indicate the two time periods that contained the most pronounced differences between the beer image and the chocolate image, chosen as a focus for all subsequent analyses.

Differences in stimulus-elicited activity as shown by the TANOVA are depicted in Figure 3. There were prominent differences between both beer and chocolate pictures, and the neutral grey square. These differences started approximately 90 ms after picture onset and persisted throughout the image-viewing period. The early onset indicates that these differences are at least initially driven by the vast perceptual differences between the two complex images of beer and chocolate as opposed to the simple grey square. The main difference between the beer vs. grey and chocolate vs. grey contrasts is that after around 550 ms the significant differences between beer and grey square are still prominent but the differences between chocolate and grey square appear to be somewhat weaker and more intermittent.

On the other hand, beer and chocolate differ from each other in three distinct time intervals: there was an initial difference between 108 ms and 195 ms, then between 265 ms and 334 ms, and finally a set of later, prolonged differences that arose at 654 ms and were intermittently present until 1357 ms.

### Event-related potentials (ERPs)

**Figure 4:**
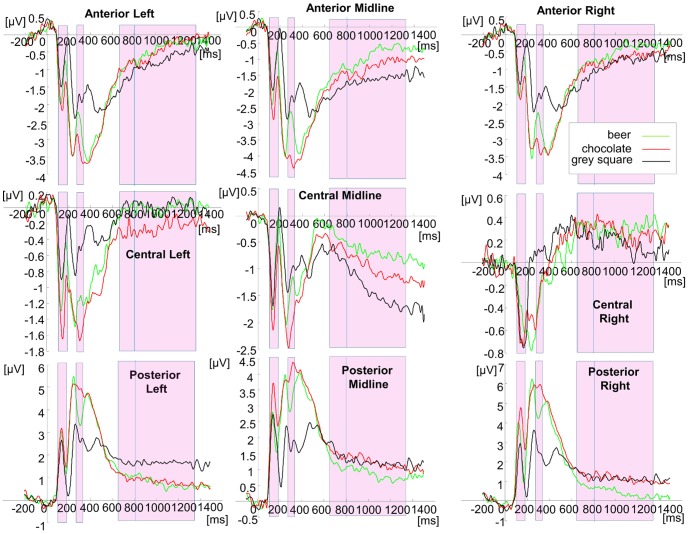
ERPs at anterior, central and posterior sites on the left, midline and the right. EEG analysis: ERPs at anterior, central and posterior sites on the left, midline and the right. Note that different voltage scales were used at different electrodes. The coloured squares indicate periods of activity indicated as revealing differences between stimuli by the TANOVA results (see Figure 3). Activity in the later period (654–1357 ms) was selected for further analysis. This window is split by the blue line to indicate the division between the earlier segment (up to 800 ms), which can be considered as part of the P300, and the later segment of activity, which is a slow potential.

ERPs are depicted in Figure 4. A 3 (picture type: beer, chocolate, neutral) x 3 (electrode laterality: left, midline, right) x 3 (electrode position: anterior, central, posterior) repeated measures ANOVA was performed on mean amplitudes in the time windows of significant differences indicated by the TANOVA in the later time window (654–1357 ms). The later window suggests modulation of ERP components that we expected to be modulated by picture type, i.e. the P300 (654 to 800 ms) and SP (800 ms and beyond).

Visual inspection of the ERPs in Figure 4 from midline central sites (those sites which traditionally show the P300 and SP in response to drug cues in substance users; [14]) shows that the time-window of significant differences in stimulus-elicited activity encompasses the latter part of the P300 followed by the Slow Potential. Therefore, in accordance with the findings from a recent meta-analysis [15] we examined both ERP components separately.

### P300

The 3×3×3 ANOVA on the earlier time window, corresponding to the latter part of the P300 (654–800 ms) revealed that the predicted 3 way interaction between picture type, electrode laterality and electrode site was not statistically significant (F(4.85,103.63) = 145.54,p = . 054, partial η^2^ = .07). The main effect of picture type was also not significant (F(2,60) = 2.82,p = .068) and neither was the picture type x electrode laterality interaction (F(4,120) = 2.12,p = .082). However, the picture type x electrode site interaction was statistically significant F(2.13,64.02) = 3.26,p = .042, partial η^2^ = .10). In order to explore this interaction, we conducted 3 post-hoc ANOVAs, one at each site, reducing the p value to .0167. None of these post-hoc tests revealed significant main effect of picture type. Therefore, no reliable differences between picture types were found in the late part of the P3 window.

When we re-ran this analysis with the addition of group as a further between-subjects factor, this revealed a significant interaction between group, picture type and electrode laterality (F(4, 108) = 2.60,p = .04, partial η^2^ = .08). In order to explore this interaction, we conducted 3 post-hoc ANOVAs, one at each laterality, reducing the p value to .0167. We found a significant interaction of picture type and group at left sites (F(2,54) = 4.47,p = 0.0160, partial η^2^ = .14). We attempted to further deconstruct this interaction with Bonferroni-corrected one-way ANOVAs for each picture type on the left, but none of these were significant.

### Slow Potentials

The same 3×3×3 way ANOVA was performed using the time interval representing the slow potential (801–1357 ms). This revealed a significant 3 way interaction (F(4.92,147.43) = 3.78,p = .0032, partial η^2^ = .12). Post-hoc analyses are presented in Table 1.

**Table 1:** Planned comparisons between ERP amplitudes at nine electrode sites in the SP time window (801–1357 ms after stimulus onset).

Electrode site	Main effect of picture type	Beer vs. chocolate	Chocolate vs. grey	Beer vs. grey
Left anterior	F(2,60) = 1.05, p>.1	/	/	/
Left central	F(2, 60) = 3.55, p = .035 (equivalent to .315)	/	/	/
Left posterior	F(2, 60) = 9.22, p = .00012 (equivalent to .0011)	p>.1	**p = .** **000351 (equivalent to .0032; less positive for chocolate)**	**p = .00115 (equivalent to .0104; less positive for beer)**
Midline anterior	F(2,60) = 5.14, p = .009 (equivalent to p = .081)	/	/	/
Midline central	**F(2,60) = 12.80,p = .000023 (equivalent to .00021)**	p = .0298 (equivalent to .268)	p = .011 (equivalent to .099)	**p = .00002 (equivalent to .00018; more positive for beer)**
Midline posterior	**F(2,60) = 1.61,p = >.1**	/	/	/
Right anterior	F(2,60) = 1.17, p>.1	/	/	/
Right central	**F(1.53,45.89) = 0.99, p>.1**	/	/	/
Right posterior	**F(2,60) = 7.87, p = .** **00092 (equivalent to .0083)**	**p = .00026 (equivalent to .0023; less positive for beer)**	p>.1	**p = .003731 (equivalent to .0336; less positive for beer)**

Footnote: Significant differences are indicated in bold. Alpha levels were Bonferroni-corrected for the number of post-hoc ANOVAs (9) and t-tests (9) that were conducted from p = .05 to p = .0056. For comparison of p values with standard p values of significance (.05, .01, .005, .001), the equivalent p value is given in the table, reached by multiplying the actual p value by the number of comparisons.

As can be seen from Table 1, the SP at midline sites was more positive for beer than for the neutral stimulus, as predicted. Generally, central activity shows a tendency for increased positivity for beer, with an increased negativity for beer at posterior sites, in line with the dipole model of EEG activity generation. Sometimes these effects were observed against the neutral stimulus (midline central), and sometimes against both chocolate and the neutral stimulus (right posterior sites). Activity elicited at left posterior sites differs from the neutral stimulus for both chocolate and beer images.

When we re-ran this analysis with the addition of group as a further between-subjects factor, this revealed no significant main effects or interactions. Therefore, for both P300 and Slow Potentials, individual differences in hazardous drinking (based on AUDIT scores) did not appear to moderate electrophysiological responses to the pictures.

### Associations between electrophysiological responses behavioral PIT effects, and individual differences in alcohol use

We performed Pearson correlations (two-tailed, Bonferroni-corrected) to investigate associations between electrophysiological responses to alcohol pictures, behavioral PIT effects, and individual differences in alcohol consumption, hazardous drinking, and craving. Differential SP amplitudes at electrode sites that revealed significant differences between the different types of pictures (see above) were correlated with the corresponding behavioral PIT effect, and with alcohol use indices. After correcting for multiple comparisons (12 comparisons, with p value reduced from .05 to .0042), all correlations between the SP amplitude difference and the corresponding behavioral PIT effect, and the correlations between these values and scores on the DAQ and AUDIT, and weekly alcohol consumption, were not statistically significant.

## Discussion

In this study, social drinkers performed a Pavlovian to instrumental transfer task that measured the influence of beer and chocolate pictures on their instrumental responding for beer and chocolate reinforcers, whilst EEG was recorded. The behavioral data indicated a PIT effect, as presentation of a noncontingent beer picture increased instrumental responses for beer reinforcers whereas presentation of a noncontingent chocolate picture increased instrumental responses for chocolate reinforcers. Therefore, our first hypothesis was supported, and this is the first demonstration of such PIT effects produced by alcohol cues in social drinkers, although it is consistent with comparable findings obtained from tobacco smokers [9].

Based on a recent meta-analysis [15], our second hypothesis was that alcohol-related and chocolate pictures would evoke distinct patterns of electrophysiological responses, in particular enhanced P300 and Slow Potentials in comparison to a neutral stimulus (a grey square). Our data provide only partial support for this hypothesis. Differences between different types of pictures were much more pronounced for slow potentials than for the P300, as one might expect [37], [38]. However the overall pattern was mixed, and there were no consistent differences between the rewarding stimuli (alcohol and chocolate pictures) and the neutral grey square at different electrode sites. Consideration of methodological differences between the tasks used in the studies included in Littel et al.'s [15] meta-analysis versus our own task may explain why we failed to detect the predicted effects. Most prior studies used an oddball task in which participants were not required to make a manual response to most of the stimuli that were presented. Whereas, in our task, electrophysiological activity was recorded as participants viewed the pictures and were awaiting a prompt to make their instrumental response. It is well known that P300 amplitude effects tend to be blunted if participants are engaged in more complex paradigms [39] which may explain our unexpected findings. Furthermore, our stimuli were also very different from those used in the previous studies reviewed by [15]. In our study, we repeatedly presented three images, 100 times each over the course of the main task, and these images were simple and relatively small pictures of a chocolate bar, a bottle of beer or a blank grey square. Previous studies used more complex drug-related pictures and included a larger number of different pictures [40]. A goal for future research is to develop a more sensitive PIT paradigm that can detect differential ERPs in response to pictorial cues, because the paradigm used in our study may have been insensitive.

Consideration of characteristics of our sample suggests an alternative explanation for the weak ERPs to alcohol cues that we observed in our study. Littel et al.'s [15] meta-analysis included studies that tested abusers of several different substances, and there was only one study of alcohol-dependent participants. The participants in our study were social drinkers with no history of alcohol dependence. Two previous studies, neither of which were included in Littel et al.'s (2012) meta-analysis, [41], [17] demonstrated increased amplitude of P300 in response to alcohol-cues (versus neutral cues) in social drinkers. However, careful reading of those papers suggests that enhanced P300 to alcohol cues may not be particularly robust in social drinkers. In one study [41], enhanced P300 to alcohol cues was only seen in a subgroup of drinkers who had low sensitivity to the effects of alcohol, a variable that we did not measure in the present study. In the Herrmann et al. [17] study, enhanced P300 to alcohol cues was only seen in heavy social drinkers (not social drinkers as a whole), and even this was limited to frontal-midline electrode sites. In that study, the amplitude of P300 was not enhanced at central or parietal sites where P300 is usually most prominent [42]. Even in samples of alcohol-dependent samples, enhanced P300 to alcohol cues is not consistently seen [43]–[45]. Researchers interested in studying the electrophysiological correlates of PIT effects in addiction are advised to study different populations, such as tobacco smokers or cocaine users, in whom ERP reactivity to drug-related cues appears to be more robust [15]. We also advise that participants use more established methodologies such as the oddball task [15] to capture ERPs in response to drug-related cues, as opposed to the modified task that we used in the present study.

Our third hypothesis related to the inter-relationships between behavioral PIT effects and the amplitudes of P300 and SP in response to the beer and chocolate pictures. We predicted that amplitudes of P300 and SP in response to beer cues (relative to their amplitudes in response to chocolate or neutral cues) would be positively correlated with the effect of those beer cues on instrumental responding for beer, i.e., behavioral PIT effects. However, all of these correlations failed to reach statistical significance. We also failed to find any significant association between individual differences in drinking habits, hazardous drinking (scores on the AUDIT) alcohol craving (scores on the DAQ), and ERP indices of cue reactivity. Furthermore, when we repeated our analyses after splitting participants into those with relatively high vs. low scores on the AUDIT (based on a median split), these analyses did not suggest that individual differences in AUDIT scores moderated the strength of behavioral PIT effects or ERPs to alcohol pictures during the PIT task. Therefore our fourth hypothesis was also rejected. The lack of association between behavioral PIT effects and individual differences in drinking habits or hazardous drinking parallels similar findings reported in cigarette smokers [7], [9]. In these studies the overall level of instrumental responding for tobacco was correlated with individual differences in nicotine dependence, but the magnitude of tobacco PIT effects was not. As discussed in the Introduction, this is consistent with the notion that PIT effects are evoked automatically by conditioned stimuli, irrespective of the level of dependence or motivational state at the time.

The absence of significant correlations between ERP indices of alcohol cue reactivity and individual differences in AUDIT scores is perhaps unsurprising given that the robust P300 and SP in response to alcohol cues that we had expected to find, did not materialize. Our inconclusive findings regarding ERP reactivity to alcohol cues are consistent with the previous studies of social drinkers and alcoholics, as discussed above. Characteristics of our sample may also explain our findings, particularly the lack of associations between AUDIT scores, ERP reactivity, and the overall level of instrumental responding for alcohol. The vast majority (81%) of our participants scored above 8 on the AUDIT and could therefore be classed as ‘hazardous drinkers’. Furthermore, the maximum AUDIT score was 23, which means that this variable had a restricted range (81% of participants had an AUDIT score between 8 and 23, and the possible range of scores on the AUDIT ranges from 0 to 40). Furthermore, because our sample was small we were unable to use sophisticated statistical techniques to explore how between-subject variables may moderate responses to within-subject experimental manipulations. Future studies should aim to recruit larger samples of participants with a wider range of drinking habits, particularly lighter or more infrequent drinkers, in order to more comprehensively investigate the relationships between hazardous drinking, instrumental responding for alcohol and ERPs to alcohol pictures. However, it is important to point out that alcohol abstainers would not be suitable for inclusion in studies such as this, as there is no reason to believe that they would ever make instrumental responses in pursuit of alcohol and indeed it would be unethical to even attempt this.

Finally, two features of the PIT effect may have been crucial in our failure to detect EEG correlates of behavioral PIT effects. First, the PIT effect appears to be mediated by explicit outcome expectancies, which paradoxically, are decoupled from the current motivational value of the outcome [46]. On this basis, it is perhaps unsurprising that behavioral PIT effects were not correlated with those ERP components that have been linked to motivational properties of drug cues, namely the P300 and SP [13]. Second, human fMRI [11], [44] and animal lesion [45], [46] studies are remarkably consistent in showing that the specific PIT effect for non-drug rewards is mediated by the striatum, amygdala, orbitofrontal cortex and mediodorsal thalamus. Although EEG is capable of detecting activity in at least some of these regions [47] it is notable that the paradigm used in the present study was very different from the PIT paradigm used in the fMRI studies [13], [48]. In order to resolve this issue, future studies attempt to obtain PIT effects in the fMRI scanner using our paradigm, or conduct an ERP study with a paradigm more closely matched to those used in previous studies such as [48].

In conclusion, this is the first study to investigate Pavlovian to instrumental transfer effects evoked by alcohol and chocolate cues, and their electrophysiological correlates, in a sample of social drinkers who regularly consumed chocolate. While we observed the behavioral PIT effects that we predicted, analysis of P300 and Slow Potential event-related potentials did not reveal the robust reactivity to alcohol cues that we had predicted. This may reflect a limitation of the task or the stimuli that we used, or it may be attributable to the characteristics of our sample.

## Supporting Information

Figure S1 **Topographical segmentation of EEG data.** (a) The full time-course of topographical changes after stimulus presentation determined by a clustering analysis of grand-mean data for the three experimental conditions. This segmentation was characterized by 6 amplitude maps. The y axis depicts global field power, an indicator of response strength. Colours represent the sequence of different topographies. Each subsequent topography is presented in a different colour and marked with a different number. The coloured squares indicate periods of significant differences indicated by the TANOVA results (see Figure 3); (b) Topographies from the segmentation analysis. These are the maps that are characteristic for the period after stimulus onset. The templates are normalized GFP-weighted averages of all maps belonging to a particular data segment.
(TIFF)

Material S1 **Segmentation into topographical maps.**
(DOCX)
