# Supplementary figures and images for: Electrophysiological Responses to Alcohol Cues Are Not Associated with Pavlovian-to-Instrumental Transfer in Social Drinkers
Source: PLoS One. 2014 Apr 14;9(4):e94605. doi: 10.1371/journal.pone.0094605 (PMC3986108; doi:10.1371/journal.pone.0094605)

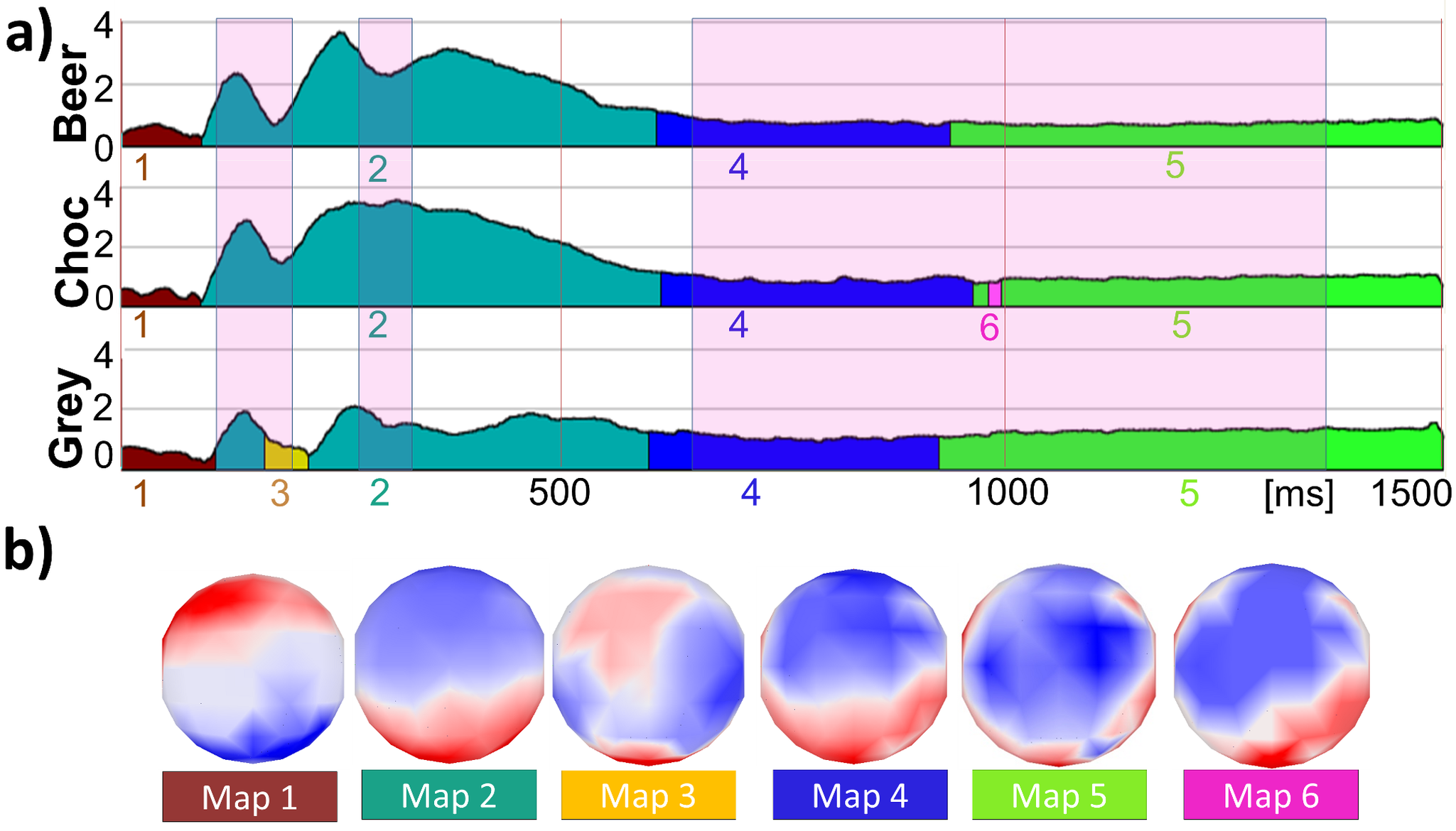

Supplement: Figure S1 — Topographical segmentation of EEG data. (a) The full time-course of topographical changes after stimulus presentation determined by a clustering analysis of grand-mean data for the three experimental conditions. This segmentation was characterized by 6 amplitude maps. The y axis depicts global field power, an indicator of response strength. Colours represent the sequence of different topographies. Each subsequent topography is presented in a different colour and marked with a different number. The coloured squares indicate periods of significant differences indicated by the TANOVA results (see Figure 3); (b) Topographies from the segmentation analysis. These are the maps that are characteristic for the period after stimulus onset. The templates are normalized GFP-weighted averages of all maps belonging to a particular data segment. (TIFF) [file pone.0094605.s001.tif]
