# Supplementary material for: Electrophysiological Responses to Alcohol Cues Are Not Associated with Pavlovian-to-Instrumental Transfer in Social Drinkers
Source: PLoS One. 2014 Apr 14;9(4):e94605. doi: 10.1371/journal.pone.0094605 (PMC3986108; doi:10.1371/journal.pone.0094605)
Supplement: Material S1 — Segmentation into topographical maps. (DOCX) [file pone.0094605.s002.docx]

Supplementary Material:

Segmentation into topographical maps

Methods:

Since TANOVA is sensitive to the difference in the temporal alternation of topographic maps (i.e., amplitude differences across time), segmentation of the EEG waveform into topographical maps was performed to see if the topographies that occurred within the time-periods indicated as significantly different by TANOVA were indeed substantially different from each other. If the difference found by the TANOVA is due to activation of different underlying generators, different maps should characterize the responses to the three different types of cues (beer, chocolate, and neutral). On the other hand, if the difference was caused by the variation in the strength of activation in the same generators over time, the same dominant topographies should be revealed by the segmentation analysis.

Segmentation was performed using a hierarchical agglomerative cluster analysis of grand-mean waveforms of activity from 0 to 1500 ms after stimulus onset, for the three picture types. This clustering approach operates in a bottom-up manner: the number of clusters is initially set to a relatively large user-selected value (here, 30 clusters) and progressively diminishes, by iteratively removing the clusters with the lowest global explained variance and assigning their maps to those surviving clusters with which they have the highest spatial correlation. The procedure is completed when only one cluster remains. The optimal solution is then chosen among the range of possible solutions such that it contains a set of clusters that provides the best explanation for variance in EEG data topographies. To determine the number of clusters that provides an optimal solution, we used the Krzanovski–Lai criterion, whose maximum shows the point at which the highest global quality of the segmentation was achieved.

Results:

Segmentation analysis revealed a set of maps characteristic of the data, which are depicted in Figure 4. In general, the topographies were dominated by a posterior positivity and anterior negativity, as could be expected on the basis of previous research using passive viewing paradigms (e.g., [[36](#_ENREF_36)]). The topographies appear highly similar between the beer and chocolate pictures in the time-windows of significant differences indicated by the TANOVA. The topography for the grey square is somewhat different in the period falling within the earliest time window (108-195 ms), which is again unsurprising given that the image is vastly perceptually different from the images of beer and chocolate

----------------------

Insert Figure S1 about here

----------------------
